# *Chlamydia psittaci* detected at a live poultry wholesale market in central China

**DOI:** 10.1186/s12879-024-09478-8

**Published:** 2024-06-12

**Authors:** Rusheng Zhang, Huiyuan Fu, Can Luo, Zheng Huang, Ruiqing Pei, Yu Di, Caiying Zhu, Jiayi Peng, Huiqi Hu, Shan Chen, Jingfang Chen, Lamei Chen, Mingzhong Xu, Xuewen Yang, Rengui Yang

**Affiliations:** 1 Changsha Center for Disease Prevention and Control, Changsha, Hunan 410007 China; 2 Public Health College, University of South China, Hengyang, Hunan 421001 China; 3 Changsha Kaifu Disease Prevention and Control Center, Changsha, Hunan 410007 China; 4 Changsha Hospital of Traditional Chinese Medicine (Changsha Eighth Hospital), Changsha, Hunan 410125 China

**Keywords:** *Chlamydia psittaci*, Environment, Live poultry markets, Poultry

## Abstract

**Background:**

We investigated the presence of *Chlamydia psittaci* in poultry and the environment in live poultry wholesale markets in Changsha during 2021–2022 and conducted a phylogenetic analysis to understand its distribution in this market.

**Methods:**

In total, 483 samples were analyzed using real-time polymerase chain reaction and 17 *C. psittaci*-positive samples using high-throughput sequencing, BLAST similarity, and phylogenetic analysis.

**Results:**

Twenty-two out of 483 poultry and environmental samples were positive for *C. psittaci* (overall positivity rate: 4.55%) with no difference in positivity rates over 12 months. *Chlamydia psittaci* was detected at 11 sampling points (overall positivity rate: 27.5%), including chicken, duck, and pigeon/chicken/duck/goose shops, with pigeon shops having the highest positivity rate (46.67%). The highest positivity rates were found in sewage (12.5%), poultry fecal (7.43%), cage swab (6.59%), avian pharyngeal/cloacal swab (3.33%), and air (2.29%) samples. The *ompA* sequences were identified in two strains of *C. psittaci*, which were determined to bear genotype B using phylogenetic analysis. Thus, during monitoring, *C. psittaci* genotype B was detected in the poultry and environmental samples from the poultry wholesale market in Changsha.

**Conclusions:**

To address the potential zoonotic threat, *C. psittaci* monitoring programs in live poultry markets should be enhanced.

## Background

Psittacosis is a zoonotic disease caused by the pathogen *Chlamydia psittaci*, which was first isolated from parrots and subsequently from 460 other bird species, including pigeons, ducks, turkeys, gulls, and acacia birds. *Chlamydia psittaci* can cause lung infections in humans directly through inhalation or indirectly through contact with carrier birds or their secretions [1–5]. This disease is also clinically known as ornithosis [6]. With no specific clinical manifestations, most human patients have a sudden onset of the disease and show symptoms, including chills, fever, coughing, and chest pain, which can progress to pneumonia. However, some patients may have a slow disease onset with occult infection [4, 7]. The lack of timely diagnosis and treatment of this disease can lead to mortality.

*Chlamydia psittaci* infections in humans are well-documented [7–9]. Most reported human *C. psittaci* infection cases are attributed to close contact with poultry [10–18]. To date, the largest psittacosis outbreak was reported in the United States in August 2018, with 82 poultry processing plant workers diagnosed with *C. psittaci* infection [19]. In 2020, a *C. psittaci* outbreak involving 22 human-to-human transmission cases occurred at a duck meat processing plant in Shandong, China [20]. In September 2019, an outbreak of clustered human *C. psittaci* infection occurred in Changsha, with all ten cases involving vendors selling poultry in the live poultry market (LPM); however, *C. psittaci* was not detected in the market environment [21].

*Chlamydia psittaci* can be transmitted across species via various routes, including direct contact with infected birds, indirect contact with objects contaminated with the bacterium, and inhalation or ingestion of aerosols or water contaminated with *C. psittaci* [10, 12, 19, 21–23]. Poultry premises, including LPMs, are the primary locations where individuals come in contact with poultry. Studies have also shown that environmental contamination in LPMs is a key factor in the transmission of infections to humans. For instance, when avian influenza subtype H7N9 contaminates an LPM, the human populations exposed to it are at risk of infection with the subtype [24–26]. Wang et al. [27] assessed the effectiveness of various LPM interventions in reducing transmission of H7N9 virus across five annual waves during 2013–2018 in China, especially in the final wave.

Four LPM interventions led to a mean reduction of 34–98% in the daily number of infections in wave 5. Notably, permanent closure resulted in the most effective reduction in human infection with the H7N9 virus, followed by long-period, short-period, and recursive closures in wave 5 [27].

As psittacosis is not classified as a notifiable infectious disease in China, no unified monitoring and reporting system has been established for cases of human infection and environmental contamination caused by *C. psittaci* in LPMs. However, in recent years, data from epidemiologic investigations have shown that environmental exposure to *C. psittaci* poses a significant risk factor for the onset of the corresponding zoonotic disease in humans [28, 29], implying the need to monitor these markets for early intervention.

Therefore, in this study, we selected a sizeable live poultry wholesale market (LPWM) in Changsha, a city in central China, to survey *C. psittaci* in poultry and the environment.

## Methods

### LPWM sampling points

**Fig. 1:**
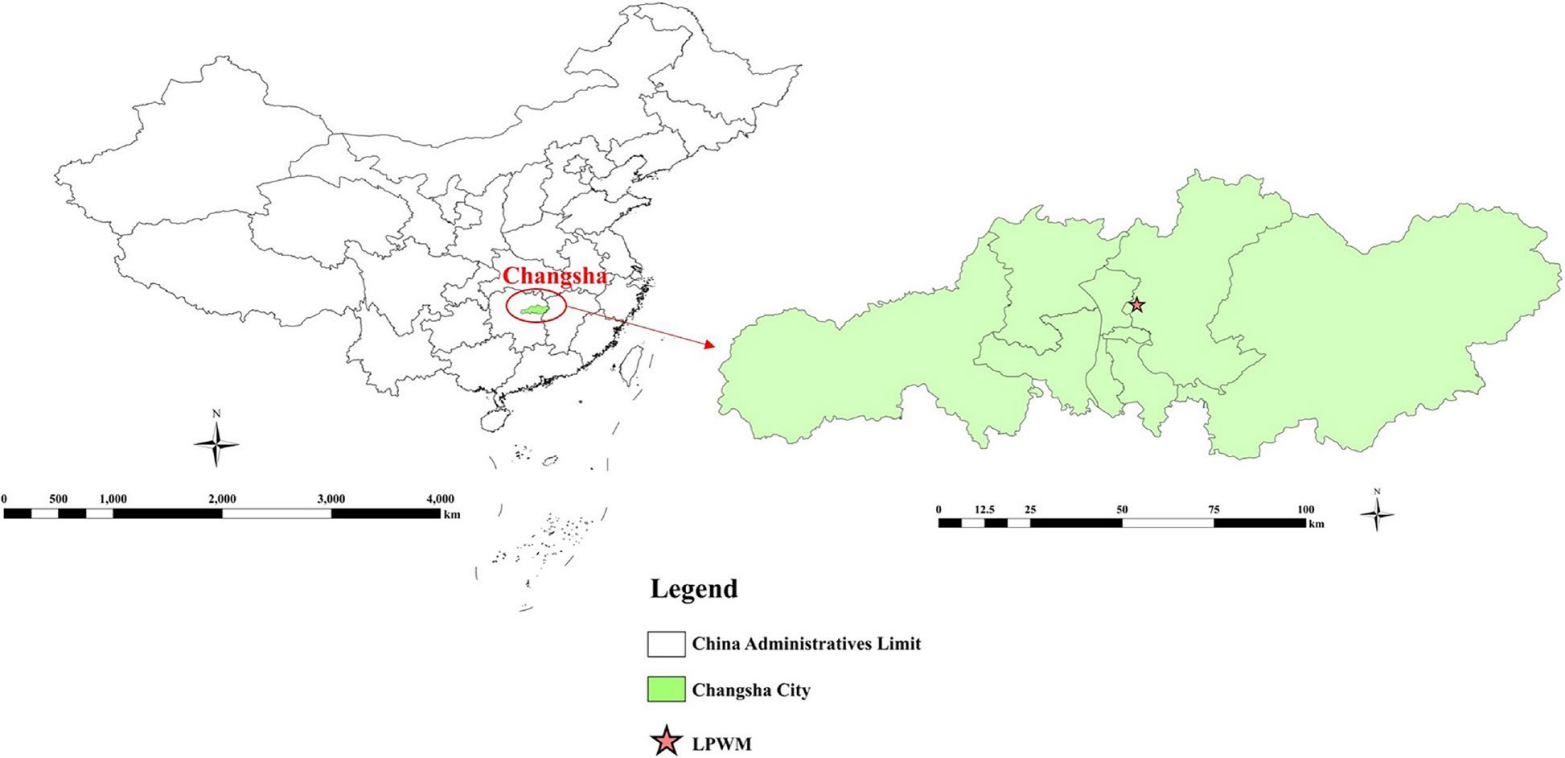
Geolocation of the live poultry wholesale market in Changsha city from China

Changsha (27°51ʹ–28°41ʹ N, 111°53ʹ–114°15ʹ E) is the capital city of Hunan Province, China. The sampling site selected in the present study was an LPWM supplying approximately 30,000 live poultry of various types, originating from all over the country, such as pigeons, local chickens, Luosi chickens, spot-billed ducks, Muscovy ducks, Peking ducks, and black and brown geese. The LPWM supplies live poultry to small- and medium-sized local live poultry (farmer) markets on a wholesale basis. The wholesale market consists of two levels. The upper level contains shops that sell mainly poultry, including pigeons and chickens, whereas the lower level contains shops that sell waterfowl, including ducks and geese. The market consists of 56 shops that can be further divided into large shops, each covering an area of approximately 300 m^2^ and selling approximately 1,500–2,000 birds per day, and small shops, each covering an area of approximately 80 m^2^ and selling approximately 300–400 birds per day. The designated operating hours for these shops span from 22:00 to 11:00 the next day, and unsold poultry is collectively culled on the same day. The LPWM sampling locations are shown in Fig. 1.

### LPWM poultry and environmental sample collection

Over a 12-month period (2021–2022), five shops were randomly selected for sampling every 2 months. Sampling was conducted between 09:00 and 12:00. Poultry (pharyngeal/ cloacal swabs and poultry feces) and environmental samples (air, sewage, water used for washing slaughtered poultry, poultry drinking water, and cage swabs) were collected using General Bacterial Sampling kits (Yocon Biotechnology, Beijing, China). The kits also include a sampling solution, which consists of sodium chloride, potassium chloride, calcium chloride, magnesium chloride, potassium dihydrogen phosphate, disodium hydrogen phosphate, and sodium thioglycolate. The present study calculates the sample size based on the formula {N = Zα^2^ × [P × (1 − P)]/d^2^, α = 0.05, Zα = 1.96, d = 0.05}. The prevalence of *C. psittaci* in pigeon feces is *p* = 5.01% in Jilin Province, China [28]. According to the formula, the minimum sampling amount is *N* = 73. Considering sampling errors, market size, and sample types, the sample size has been expanded to 480 samples for the entire research plan. In total, 483 LPWM poultry and environmental samples were collected, consisting of 181 poultry samples (60 pharyngeal/cloacal swabs and 121 fecal samples) and 302 environmental samples (87 air samples, 24 sewage samples, 53 wash water samples, 47 drinking water samples, and 91 cage swabs).

#### Collection of avian pharyngeal/cloacal swabs

Polypropylene-tipped swabs (Yocon Biotechnology) were used to wipe the avian pharynx and cloaca 3–5 times each. The swabs were transferred to sampling tubes containing 3.5 mL of the sampling solution. Avian pharyngeal/cloacal swab samples were collected from each shop at each time point.

#### Collection of poultry fecal samples

Approximately 3–5 g of poultry fecal samples were collected from poultry housing and transferred into sampling tubes containing 3.5 mL of the sampling solution. Two samples were collected from each shop at each time point.

#### Collection of air samples

Two locations in each shop, next to the poultry housing and the feather removal machine, were selected as air sampling sites, with each sampling site located approximately 1.5 m above ground. An air sampler (Coriolis µ, Bertin Technologies, Aix-en-Provence, France) was used to collect air samples for 10 min at 200 L/min at each sampling site, directly into a small Erlenmeyer flask containing the sampling solution. Details of the collection and preservation methods are described in the literature [30] and the manufacturer’s instructions. Finally, the sampled liquid was transferred to the sampling tube, capped tightly, and labeled.

#### Collection of sewage samples

Disposable transfer pipettes (Huankai Microbial Science and Technology Co., Ltd. Guangdong, China) were used to collect 10 mL samples of sewage on the ground or in the sewers at each sampling point. The samples were transferred into a 15 mL sampling tube with an external screw cap. One sample was collected at each time point.

#### Collection of poultry drinking water and water used for washing slaughtered poultry samples

Disposable transfer pipettes (Huankai Microbial) were used to collect 10 mL of the sample from the poultry drinking water tank and another 10 mL of wastewater from the poultry washing and slaughtering station. The samples were transferred into sterilized 15 mL sampling tubes with external screw caps. One sample each of poultry drinking and wash water was collected from each shop at each time point.

All samples were transported at 4 °C, within 4 h of collection, to the laboratory of Changsha Center for Disease Prevention and Control, Changsha, China.

### Polymerase chain reaction tests for *C. psittaci* in LPWM samples

Nucleic acid extraction from poultry and environmental samples was carried out using nucleic acid extraction kits (Tianlong Science and Technology, Xi’an, China). Briefly, 200 µL of each poultry and environmental sample was mixed well and added to the lysis buffer. Nucleic acids were automatically extracted from the samples using a magnetic bead system and corresponding reagent kits (Tianlong Science and Technology, Xi’an, China). The extracted nucleic acid samples were tested for *C. psittaci* using a real-time fluorescence PCR nucleic acid detection kit (Zijian Biotechnology, Shenzhen, China).

The reaction mixture comprised the following constituents: 19.0 µL of CPS PCR Buffer, 1.0 µL of CPS Enzyme Mix, 5.0 µL of template DNA, 5.0 µL of negative control, and 5.0 µL of positive control. The reaction conditions were as follows: 50 °C for 2 min, 95 °C for 3 min, and 40 cycles at 95 °C for 5 s and 60 °C for 45. The results were interpreted according to the manufacturer’s instructions.

### Sequencing of *C. psittaci* DNA in LPWM samples

Based on the application of real-time fluorescence PCR for the detection of samples with CT values > 30 and the subsequent challenges in obtaining complete genome sequences, 17 poultry and environmental samples collected from an LPWM in Changsha during 2021–2022 that were *C. psittaci*-positive by real-time PCR (CT value < 30) were subjected to DNA extraction using the PureLink Genomic DNA Kits (Thermo Fisher Scientific, Carlsbad, CA, USA). The extracted DNA was amplified with PCR using the primers for the *ompA* gene (Cp-F1: 5ʹ-GTGAATTCTGATGCGAACGG-3ʹ; Cp-R1: 5ʹ-CTTGCCTGTAGGGAACCCAG-3ʹ). The reaction mixture comprised the following constituents: 12.5 µL of DreamTaq Green PCR Master Mix (2×) (Thermo Fisher Scientific, USA); 1.0 µL of Cp-F1; 1.0 µL of Cp-R1; 1.0 µL of template DNA; and 9.5 µL nuclease-free water. The reaction conditions were as follows: 95 °C for 2 min and 40 cycles at 95 °C for 30 s, 55 °C for 30 s, and 72 °C for 1 min, followed by incubation at 72 °C for 5 min. The *C. psittaci*-positive samples presenting the target band (1279 bp) were subjected to high-throughput nucleotide sequencing using the Illumina MiSeq sequencing system and kits (Illumina, San Diego, CA, USA).

### Basic local alignment search tool comparison and molecular evolution analysis of *C. psittaci ompA* gene

Sequences of the *ompA* gene obtained by high-throughput nucleotide sequencing were analyzed and compared using BLAST. The amino acid sequences of *C. psittaci* downloaded from GenBank and the *C. psittaci* strains obtained in this study were compared using ClustalW in MEGA 6 software (https://www.megasoftware.net/). A molecular evolutionary tree was constructed using the maximum likelihood method and tested using a bootstrap test (1,000 iterations).

### Statistical analysis

SPSS software (version 24.0; IBM SPSS, Armonk, NY, USA) was used for statistical analysis. Comparisons between positivity rates were performed using Fisher’s exact χ^2^ test. *P* values < 0.05 indicated statistical significance.

## Results

### LPWM poultry and environmental *C. psittaci* monitoring at different time-points

We found that 22 of 483 poultry and environmental samples collected from the LPWM during 2021–2022 tested positive for *C. psittaci*, with an overall positivity rate of 4.55%. The respective positivity rates in 2021 and 2022 were 3.47% (6/173) and 5.16% (16/310). *Chlamydia psittaci* was detected in samples collected in 83.33% of the sampling months (10/12), with samples collected in September 2022 having the highest positivity rate (10.64%), followed by samples collected in April 2022 (9.30%) and January 2021 (8.00%). Statistical analysis showed no differences in *C. psittaci*-positivity rates between samples collected in different months. The results are summarized in Table 1.

**Table 1:** PCR test results of *Chlamydia psittaci* in poultry and environmental samples from a live poultry wholesale market in Changsha at different time points from 2021–2022

Year	Number of positive / total number of samples (%, 95%CI)	Number of positive / total number of samples by month (%, 95%CI)										
		Jan	Feb	Mar	Apr	May	Jun	Aug	Sep	Nov	χ^2^	*P*-value
2021	6/173(3.47,0.71–6.22)	2/25(8.00,1.00–26.00)	2/30(6.67,1.00–22.00)	1/25(4.00,0.00–20.00)	1/32(3.13,0.00–16.00)	0/30(0.00,0.00–12.00)	0/31(0.00,0.00–11.00)	—	—	—		
2022	16/310(5.16,2.68–7.64)	—	—	—	4/43(9.30,3.00–22.00)	1/79(1.27,0.00-3.79)	1/47(2.13,0.00–11.00)	3/47(6.38,1.00–18.00)	5/47(10.64,4.00–23.00)	2/47(4.26,1.00–15.00)		
Total	22/483(4.55,2.69–6.42)	2/25(8.00,1.00–26.00)	2/30(6.67,1.00–22.00)	1/25(4.00,0.00–20.00)	5/75(6.67,0.89–12.44)	1/109(0.92,0.00-2.74)	1/78(1.28,0.00-3.83)	3/47(6.38,1.00–18.00)	5/47(10.64,4.00–23.00)	2/47(4.26,1.00–15.00)	12.55	0.06

Comparisons of positivity rates across months were conducted using the Fisher’s exact χ^2^ test

“—” indicates undetected data

### LPWM poultry and environmental *C. psittaci* monitoring at different sampling points

**Fig. 2:**
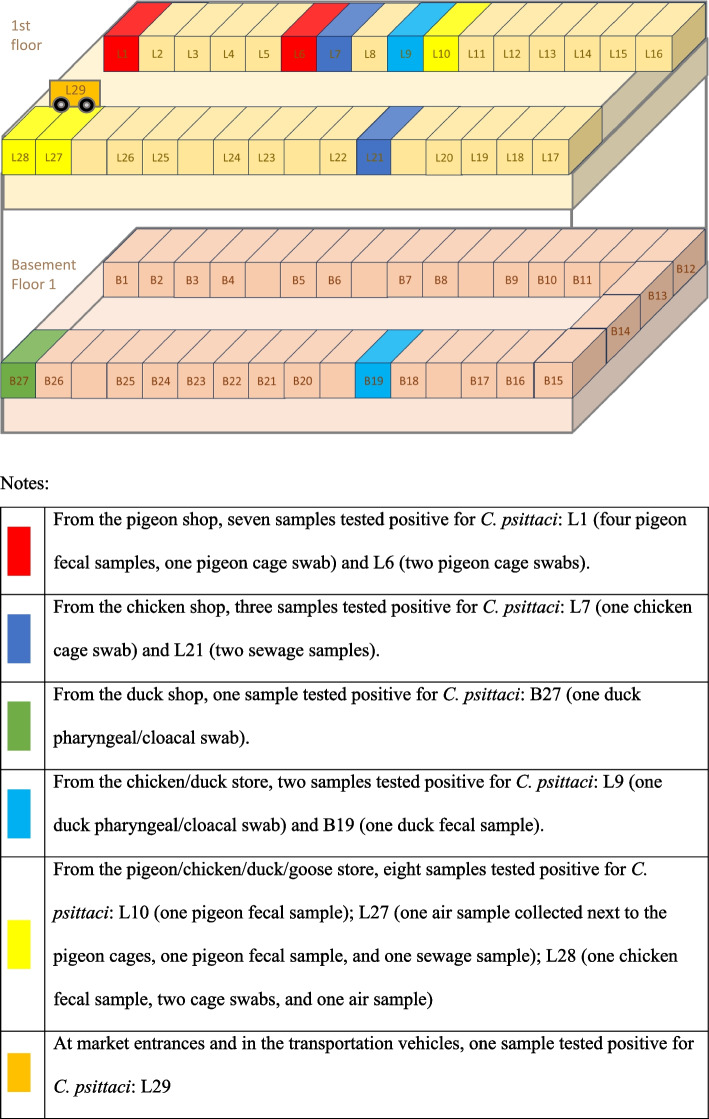
Schematic of *Chlamydia psittaci*-positive sampling sites in the live poultry wholesale market. Notes

The 40 sampling points mainly consisted of pigeon, chicken, duck, chicken/duck, pigeon/chicken/duck/goose shops, market entrances, transportation vehicles, and other shops that sell animals (such as goats, sheep, and yellow cattle). Based on the sequential sampling principle, 483 samples were collected in 70 batches from the total sampling points (*n* = 40). *Chlamydia psittaci* was detected in the samples collected from 11 sampling points (positivity rate of 27.50%). *Chlamydia psittaci*-positivity rates differed significantly (*P* < 0.01) among samples collected from different sampling points, with the samples from the pigeon shop having the highest rate (46.67%). Except for shops that sold other animals, *C. psittaci* was detected in samples collected from all other sampling points, such as chicken and duck shops and the pigeon/chicken/duck/goose shops. The results are presented in Table 2; Fig. 2.

**Table 2:** *Chlamydia psittaci* PCR test results of samples collected from different sampling points at the Changsha live poultry wholesale market, 2021–2022

Year	Number of positive / total number of samples (%, 95%CI)	Number of positive / total number of samples at each sampling point (%, 95%CI)								
		Pigeon Shop	Chicken Shop	Duck Shop	Chicken/Duck Store	Pigeon/Chicken/ Duck/Goose Store	Market entrances and goods transportation vehicles	Stores selling other animals	χ^2^	*P*-value
2021	6/173(3.47,0.71–6.22)	—	3/67(4.48,0.00-9.56)	0/12(0.00,0.00–26.00)	1/34(2.94,0.00–15.00)	2/60(3.33,0.00-8.01)	—	—		
2022	16/310(5.16,2.68–7.64)	7/15(46.67,21.00–73.00)	0/114(0.00,0.00-1.33)	1/9(11.11,0.00–48.00)	1/41(2.44,0.00–13.00)	6/103(5.83,1.23–10.43)	1/13(7.69,0.00–36.00)	0/15(0.00,0.00–22.00)		
Total	22/483(4.55,2.69–6.42)	7/15(46.67,21.00–73.00)	3/181(1.66,0.00-3.54)	1/21(4.76,0.00–24.00)	2/75(2.67,0.00-6.40)	8/163(4.91,1.56–8.26)	1/13(7.69,0.00–36.00)	0/15(0.00,0.00–22.00)	30.21	< 0.01

Comparisons of positivity rates at different sampling points were conducted using the Fisher’s exact χ^2^ test

“—” indicates undetected data

### Monitoring results of *C. psittaci* in different poultry and environmental samples at LPWM

*Chlamydia psittaci*-positivity rates differed significantly among the sample types (*P* = 0.04). Sewage samples had the highest positivity rate (12.5%), followed by poultry fecal samples (7.44%), cage swabs (6.59%), avian pharyngeal/cloacal swabs (3.33%), and air samples (2.29%). *Chlamydia psittaci* was not detected in the water used for washing slaughtered poultry or poultry drinking water samples. The results are summarized in Table 3.

**Table 3:** Results of *Chlamydia psittaci* polymerase chain reaction tests of poultry and environmental samples from LPWM in Changsha, 2021–2022

Year	Number of positive / total number of samples (%, 95%CI)	Number of positive / total number of samples for each type of sample(%, 95%CI)							χ^2^	*P*-value
		Avian pharyngeal/ cloacal swabs	Poultry fecal samples	Air samples	Sewage samples	Samples of water used for washing slaughtered poultry	Poultry drinking water samples	Cage swabs		
2021	6/173(3.47,0.71–6.22)	—	0/29(0.00,0.00–12.00)	1/58(1.72,0.00-5.18)	3/13(23.08,5.00–54.00)	0/18(0.00,0.00–19.00)	0/26(0.00,0.00–13.00)	2/29(6.90,1.00–23.00)		
2022	16/310(5.16,2.68–7.64)	2/60(3.33,0.00-8.01)	9/92(9.78,3.60-15.97)	1/29(3.45,0.00–18.00)	0/11(0.00,0.00–28.00)	0/35(0.00,0.00–10.00)	0/21(0.00,0.00–16.00)	4/62(6.45,0.16–12.74)		
Total	22/483(4.55,2.69–6.42)	2/60(3.33,0.00-8.01)	9/121(7.44,2.70-12.18)	2/87(2.29,0.00-5.51)	3/24(12.5,3.00–32.00)	0/53(0.00,0.00–6.00)	0/47(0.00,0.00–8.00)	6/91(6.59,1.40-11.79)	11.73	0.04

Positivity rates for each sample type were compared using the Fisher’s exact test

“—” indicates undetected data

### Nucleotide sequencing and molecular evolution analysis of *C. psittaci* in the LPWM poultry and environmental samples

**Fig. 3:**
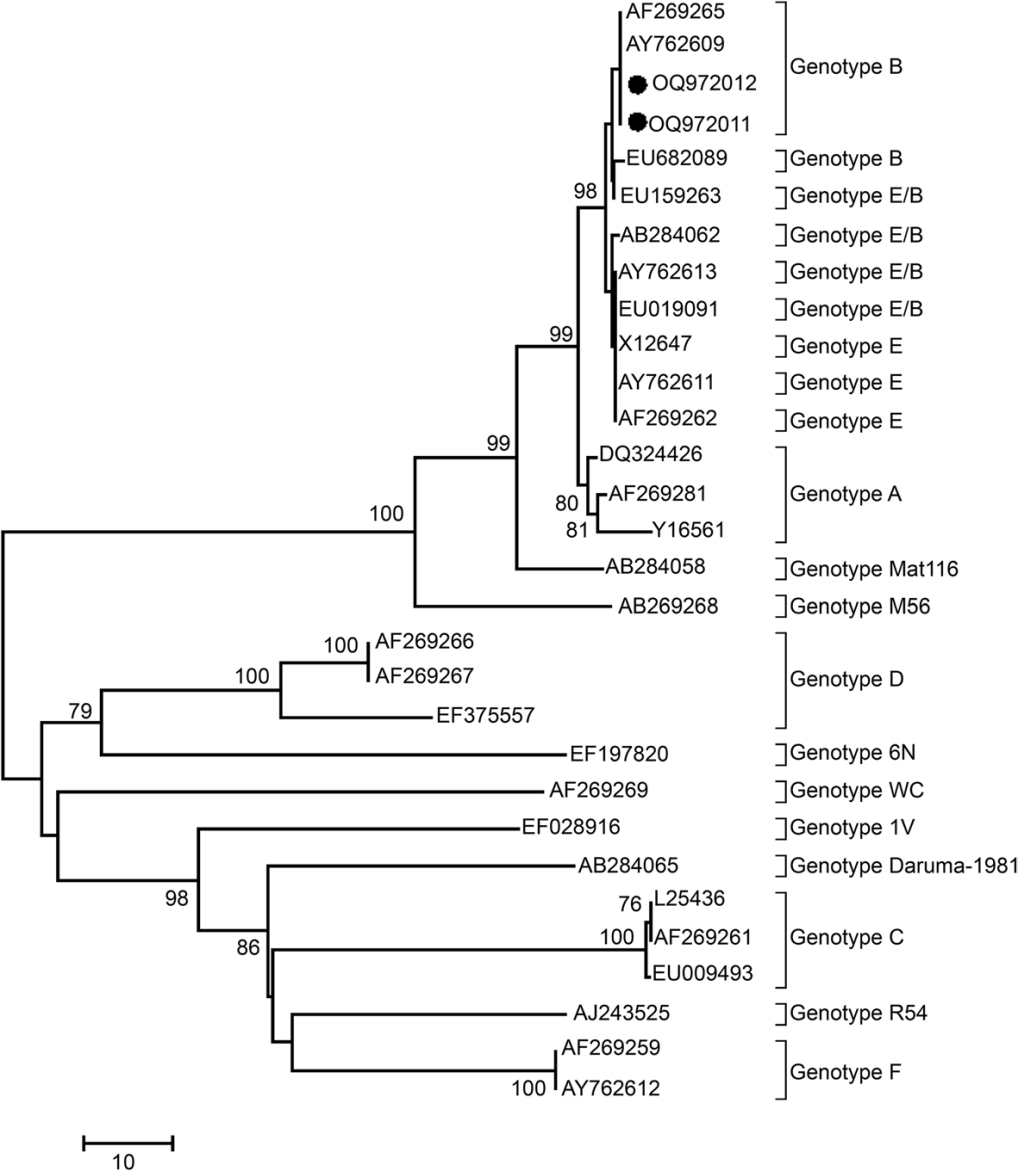
Phylogenic analysis of *Chlamydia psittaci* isolates based on the nucleotide sequence of the *ompA* gene. The *C. psittaci* strains isolated in this study are indicated by black circles

Two sequences of the *ompA* gene were successfully obtained using the Illumina MiSeq high-throughput nucleotide sequencing technique (GenBank accession numbers: OQ972011, OQ972012). BLAST similarity analysis showed that the nucleotide sequences of the two *C. psittaci* strains had the highest similarity (100%) with the *C. psittaci* strain CP3 with genotype B (GenBank accession number: CP003797.1), originating from pigeons in California, United States. Therefore, the genotypes of the two strains of *C. psittaci* were confirmed to be genotype B. Phylogenetic analysis showed that the *ompA* gene sequences of the two *C. psittaci* strains were located in the clusters of genotype B branches and closely related to the representative strains of *C. psittaci* with genotype B (GenBank accession numbers: AF269265 and AY762609, respectively), as shown in Fig. 3.

## Discussion

LPWMs mainly supply wholesale live poultry to local small- and medium-sized live poultry (farmers) markets. By monitoring *C. psittaci* in poultry and the environment of large LPWMs that supply live poultry, the status of poultry infections and environmental contamination by *C. psittaci* in local poultry markets can be evaluated to a certain extent. To the best of our knowledge, the present study is the first to conduct an environmental *C. psittaci* survey of an LPWM in China. In this study, *C. psittaci* genotype B was detected in the pigeon fecal samples obtained from LPWM in Changsha by monitoring during 2021–2022.

Recent studies have shown that many avian species are susceptible to *C. psittaci* infection [5]. For example, Liu et al. used cloacal or fecal swabs from domestic waterfowl, parrots, pigeons, and wild birds to determine the prevalence of *C. psittaci* in Taiwan during 2014–2017. They found that the prevalence of *C. psittaci* infection in waterfowl farms was as high as 34.2%. Moreover, 3.1% of samples collected from parrots were positive for *C. psittaci* of genotype A, 10.1% of samples collected from pigeons contained *C. psittaci* of genotype B, and the *C. psittaci*-positivity rate of samples collected from wild birds was 2.2% [31]. Yao et al. collected 399 pigeon fecal samples from Jilin Province, China, and found that the infection rate of *C. psittaci* in pigeons was 5.01% for all *C. psittaci* with genotype B [28].

Yin et al. [22] tested sera from Belgian and French chicken farms using ELISA for *C. psittaci*’s major outer membrane protein (MOMP). Belgian broilers, breeders, and layers had 96%, 93%, and 90% seropositivity, respectively, whereas French broilers had 91%. *Chlamydia psittaci* infections are emerging in chickens in Belgium and Northern France, posing a human psittacosis risk to chicken-processing plant employees [22]. Hulin et al. [23] confirmed high *C. psittaci* prevalence in French mule duck flocks. Environmental contamination, correlating with shedding dynamics, appears to be the main transmission pathway. High prevalence of bacteriophage Chp1, often coexisting with *Chlamydia*, suggests a key role in *C. psittaci* persistence, increasing human risk [23]. The present study showed that the *C. psittaci*-positivity rate in samples collected from an LPWM in Changsha in 2021–2022 was 4.55% (22/483) in poultry and environmental samples, 45.45% (10/22) in pigeon-related samples, 13.64% (3/22) in duck-related samples, and 9.09% (2/22) in chicken-related samples; however, it was not observed in other animal samples. The results suggest that pigeons, chickens, and ducks are the main source of environmental pollution caused by *C. psittaci* in the LPWM, and pigeons, chickens and ducks infected with *C. psittaci* pose cross-species transmission risks to human beings. *Chlamydia psittaci*-positive samples were detected in 83.33% of the sampling months (10 of 12 months), and the positivity rates of samples collected in different months did not vary significantly, suggesting the persistent presence of poultry infections and environmental contamination of *C. psittaci* in the LPWM.

Forty animal sales shops in the LPWM, including poultry shops, were selected as sampling points. The distribution map of the sampling points yielded positive samples showing that *C. psittaci* contamination was mainly found in pigeon, pigeon/chicken/duck/goose, and neighboring poultry shops. *Chlamydia psittaci* was also detected in other non-adjacent poultry shops, such as chicken and duck shops. However, other animal shops that were not spatially connected to poultry shops did not have detectable *C. psittaci*, suggesting aerosol transmission of *C. psittaci* between poultry shops that are spatially connected. For example, 5, 3, and 4 positive cases of *C. psittaci* were detected in adjacent stores L1, L27, and L28, respectively; 2 and 1 positive cases of *C. psittaci* were detected in adjacent stores L6 and L7, respectively; adjacent stores L9 and L10 both detected 1 positive case of *C. psittaci*.

In the present study, *C. psittaci*-positive poultry and environmental samples from the LPWM were predominantly fecal, cage swab, air, and sewage samples from the pigeon shop. *C. psittaci* was also detected in poultry fecal samples, duck pharyngeal/cloacal swabs, cage swabs, and air and sewage samples from other poultry (chickens and ducks) sampling sites. Therefore, in addition to the environmental samples associated with pigeon shops in the LPWM, some of the other poultry (chicken and duck) and their corresponding environmental samples were also *C. psittaci*-positive, suggesting a wide scope of LPWM poultry infection and environmental contamination of *C. psittaci*.

The findings imply that enhancement of the efficacy of cleaning and disinfecting LPWM environments is necessary. This study showed that the highest positivity rates were found in sewage (12.5%), poultry fecal (7.43%), cage swabs (6.59%), avian pharyngeal/cloacal swabs (3.33%), and air (2.29%) samples. It is recommended to centrally disinfect and discharge the sewage generated from the LPWM into the municipal sewage pipeline network, strengthen ventilation measures for the market and sales stores, and increase protective measures such as wearing gloves and masks for poultry sellers to avoid human infection with *C. psittaci* from the market environment [10].

Zhang et al. [20] reported the genotype of *C. psittaci* isolated from the human case from Shandong, China, is type A. Based on differences in the *ompA* gene, which encodes MOMP, *C. psittaci* was classified into 15 genotypes: A, B, C, D, E, F, E/B, MatI16, M56, CPX0308, WC, 6 N, 1 V, Daruma-1981, and R54, among which genotypes A to F, E/B, M56, and WC were the most common [29, 31–34]. In nature, avians are the main hosts of *C. psittaci* with genotypes A to F and E/B [32], whereas mammals host *C. psittaci* with genotypes M56 and WC [29, 33]. The pathogenicity of *C. psittaci* is genotype-dependent, with genotype A and D strains being highly virulent and causing acute infections in avian species, such as parrots and pigeons, whereas genotype A strains are commonly the culprits in human infections [31, 34]. When designing primers for nucleotide sequencing of *C. psittaci*, we prioritized enhancing the sensitivity of the primers over maximizing the specificity for *C. psittaci*. Consequently, the designed primers can identify *C. psittaci* as well as other *Chlamydia* strains, such as *C. buteonis*, *C. abortus*, and uncultured *Chlamydia*. Therefore, performing a BLAST similarity analysis on the nucleotide sequences obtained through sequencing is essential to verify the identified *Chlamydia* strains. The two *C. psittaci* strains used in this study were confirmed to be genotype B based on their *ompA* sequences. These two strains were detected in pigeon fecal samples, confirming that pigeons are susceptible to genotype B *C. psittaci* infections [28, 35, 36].

The limitation of this study was that the monitoring period for the LPWM was during the prevention and control phase of the COVID-19 outbreak. Thus, the time points for sample collection were affected, resulting in an irregular selection of sampling months. Moreover, the *C. psittaci* detected in the samples during the monitoring process was not isolated and cultured for identification. Another limitation of this study was that we did not attempt to identify other *Chlamydia* strains, such as *C*. avium, *C*. *gallinacea*, *C*. *buteonis*, or *C. abortus*.

Yin et al. reported 32 cases of human infection with *C. psittaci* in Zhejiang Province during 2020–2021; in all of these cases, individuals had a history of exposure to poultry or pigeons [18]. The present study detected *C. psittaci* in a local LPWM in Changsha, suggesting the need to develop preventive and control measures for human *C. psittaci* infection in the context of the increasing number of human *C. psittaci* infection cases. These measures involve strengthening the monitoring of *C. psittaci* in avian and public places, aimed at curbing the spread of pathogens from centralized pigeon suppliers, such as pigeon farms, to prevent *C. psittaci* infection.

## Conclusions

*Chlamydia psittaci* genotype B was detected in the poultry and environmental samples from a poultry wholesale market in central China, indicating the need to further enhance environmental monitoring and disease prevention and control of *C. psittaci* in poultry wholesale markets.

## Data Availability

The datasets generated during and/or analyzed during the current study are available in Genbank [National Center for Biotechnology Information (https://www.ncbi.nlm.nih.gov/nuccore/OQ972011,OQ972012)] [Accession Nos: OQ972011 and Q972012].

## Abbreviation

LPWM: Live poultry wholesale market
